# Double Trouble: A Rare Clinical Presentation of Bladder and Umbilical Endometriosis

**DOI:** 10.7759/cureus.98145

**Published:** 2025-11-30

**Authors:** Venkateshen Palanisamy, Sushmitha Kothapalli, Velmurugan Palaniyandi, Hariharasudhan Sekar, Sriram Krishnamoorthy

**Affiliations:** 1 Urology, Sri Ramachandra Institute of Higher Education and Research, Chennai, IND

**Keywords:** bladder endometriosis, cyclical hematuria, cyclical umbilical bleeding, endometriosis, umbilical endometriosis

## Abstract

Endometriosis is a common condition among women where tissue resembling the uterine lining grows outside the uterus. This misplaced tissue can develop in several areas, including the peritoneum, pouch of Douglas, vagina, uterosacral ligaments, rectovaginal septum, rectosigmoid junction, bladder, and umbilicus. Although endometriosis most often affects the pelvic organs, cases involving the bladder are relatively rare. The exact cause of bladder endometriosis remains uncertain, and when it occurs alongside umbilical endometriosis (Villar's nodule), it is considered an infrequent clinical presentation. We present a case report of a 42-year-old woman who presented with cyclic blood in her urine along with cyclic bleeding from her umbilicus. Her initial evaluation and examination did not provide a definitive diagnosis. An MRI of the abdomen and pelvis revealed a lesion in the umbilical area, which was suggestive of an endometrial growth. Further investigation with cystoscopy identified a polypoidal mass on the posterior bladder wall. The histopathological examination confirmed the presence of endometriosis in the umbilicus and bladder. This report presents the first documented instance of concurrent bladder and umbilical endometriosis, exploring the potential etiological factors, clinical presentation, diagnostic workup, and management options.

## Introduction

Endometriosis is one of the common gynaecological conditions characterised by the presence of tissue that closely resembles the endometrial tissue outside the uterus. The most common presentation is pelvic pain and infertility. While endometriosis primarily affects pelvic structures such as the ovaries and fallopian tubes, with advancements in diagnostic laparoscopy and imaging techniques, we have identified many other extra-pelvic sites of occurrence. Endometriotic lesions have been found in various other organ systems, including the genitourinary tract, intestine, peritoneum, and, in rare instances, even the brain [1].

The definitive diagnosis of endometriosis is confirmed by histopathological analysis, which requires identification of endometrial glandular and stromal tissue outside the uterus. Although several non-invasive methods, such as imaging studies and serum biomarkers, have been explored to aid diagnosis, their sensitivity and specificity remain low. As a result, diagnosing endometriosis is often challenging. The most definitive way to confirm the condition is through the direct visualisation and biopsy of lesions during laparoscopy or laparotomy.

One rare site of extra-pelvic endometriosis is umbilical endometriosis, also known as Villar's nodule. This condition is rare and often results from iatrogenic seeding following previous uterine surgery. However, in some cases, umbilical endometriosis occurs without any history of prior abdominal surgery or uterine procedures, making it an even rarer phenomenon [2]. Patients with this condition may experience periodic pain, cyclic bleeding from the umbilical region, and localised swelling.

Bladder endometriosis is an even rarer presentation, which affects approximately 1-2% of women diagnosed with endometriosis. It occurs when ectopic endometrial tissue is implanted into the bladder wall, leading to symptoms such as cyclical haematuria (blood in the urine during menstruation), painful urination (dysuria), and pelvic pain [3].

This report documents the first case in the literature of a 42-year-old woman diagnosed with concurrent bladder and umbilical endometriosis at a single presentation. It discusses the incidence, clinical features, diagnostic challenges, and management considerations of these rare manifestations.

## Case presentation

We present the case of a 42-year-old woman who had recurrent cyclical haematuria (blood in the urine during menstruation) and cyclical bloody discharge from her umbilicus for the past three months. The haematuria was in relation to her menstrual cycle. She had a previous history of a lower segment caesarean section (LSCS) seven years ago, along with diabetes mellitus and hypothyroidism, for which she was on regular medication.

**Figure 1:**
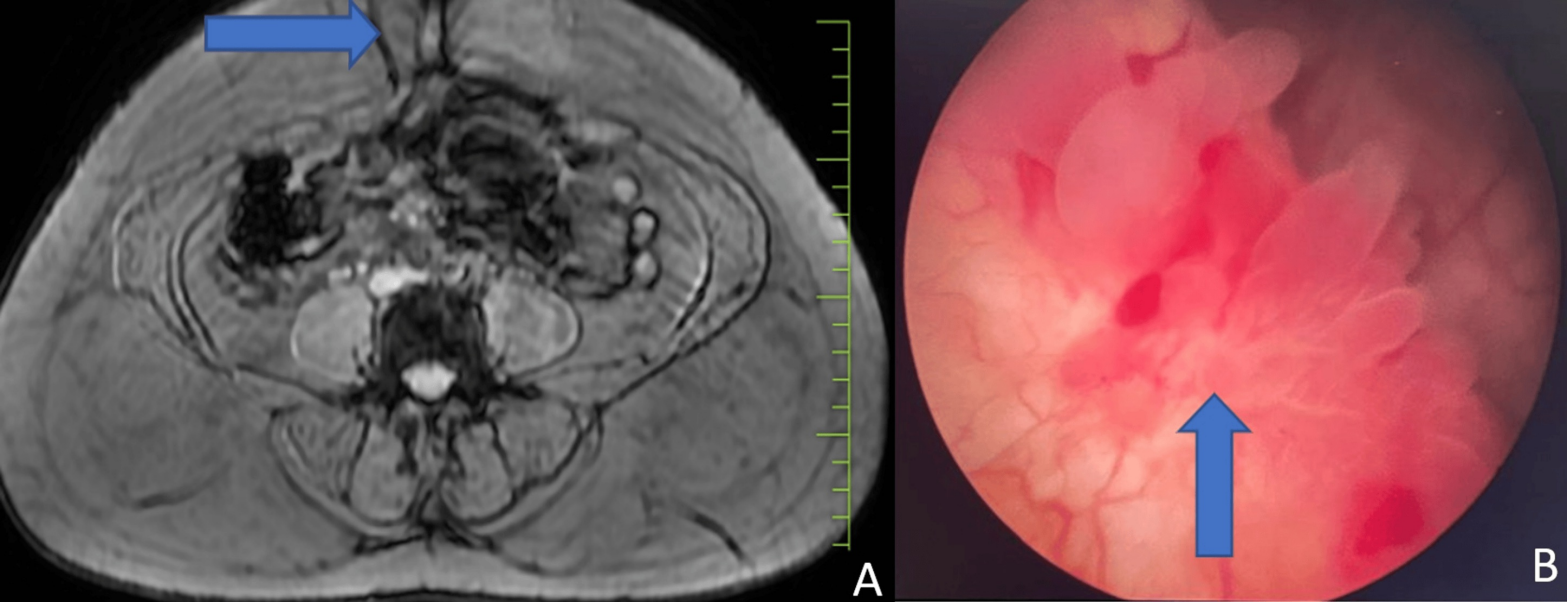
(A) MRI scan of the abdomen and pelvis, showing a T1 hyperintense lesion in the umbilical region, with a blue arrow pointing to the umbilical nodule. (B) Cystoscopy showing a 2 x 2 cm polypoidal lesion in the posterior wall of the bladder, with a blue arrow pointing to the lesion in the bladder.

On physical examination, no significant abnormalities were found. She underwent urine microscopy, which showed RBC++, and the urine culture was negative for any growth of organisms. The renal function test was within normal limits. Haemoglobin was within normal limits. The ultrasound examination of the abdomen and pelvis revealed normal results. An MRI scan of the abdomen and pelvis was performed (Figure 1A). MRI imaging showed a T1 hyperintense and T2 hypointense lesion in the umbilical region measuring 1.5 × 1.5 cm, strongly suggestive of an endometrial deposit.

**Figure 2:**
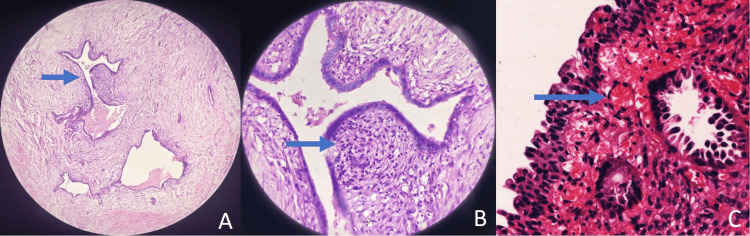
Histopathological examination of umbilical endometriosis and bladder endometriosis. (A) Histopathological examination of umbilical endometriosis, which shows findings of endometrial glands with dense cellular stroma with haematoxylin and eosin (H&E) at 10×, with the blue arrow showing the endometrial gland with stroma. (B) Histopathological examination of umbilical endometriosis shows endometrial glands with dense cellular stroma (H&E 100×; blue arrow). (C) Histopathological examination from bladder biopsy showing urothelial epithelium with an arrow showing the subepithelium and endometrial glands surrounded by haemorrhage, suggestive of endometrial endometriosis (H&E stain 400×).

Given the history of cyclical haematuria and umbilical nodule on MRI, a diagnostic cystoscopy was performed (Figure 1B). Cystoscopy revealed a polypoidal lesion on the posterior bladder wall, raising suspicion of bladder endometriosis. To confirm the diagnosis, a bladder biopsy was performed during cystoscopy, and umbilical nodule biopsies were obtained under anaesthesia. Histopathological examination confirmed the presence of endometriotic tissue in the bladder biopsy and umbilical biopsy (Figures 2A-C).

After confirming the diagnosis of both bladder and umbilical endometriosis, the patient was referred to a gynaecologist, who started her on progestin hormonal therapy to suppress the growth of ectopic endometrial tissue and manage symptoms. Over the next two months of follow-up, the patient remained symptom-free, with no further episodes of haematuria or umbilical bleeding. She is symptom-free and under regular hormonal therapy. She is on regular follow-up.

## Discussion

Endometriosis was originally described as the presence of "endometrial-like tissue" outside the uterus. Initially, it was often discovered accidentally during abdominal surgeries performed for other conditions. However, by the 1960s, it was evident that endometriosis was one of the major causes of pelvic pain and infertility, frequently needing surgical intervention. The introduction of laparoscopy in the 1980s changed its diagnosis and management, revealing that pelvic endometriosis was one of the most common causes of infertility and pain in the reproductive age.

Endometriosis is one of the common presentations in women worldwide, with an incidence of 5-10% of all women who may be symptomatic or asymptomatic. Studies report that 4.1% of women undergoing tubal ligation have asymptomatic endometriosis, and 38% of women with infertility have been diagnosed with endometriosis. However, obtaining an exact incidence is challenging since a definitive diagnosis requires laparoscopy or surgical biopsy [4].

The exact pathogenesis of endometriosis remains unclear, but research suggests it is multifactorial and arises from a combination of genetic, hormonal, immune, and environmental factors. One of the most accepted theories is retrograde menstruation, which suggests that menstrual blood flows backwards through the fallopian tubes, allowing endometrial tissue to implant and grow outside the uterus. Other theories are related to metaplasia, oxidative stress, immune dysfunction, and genetic predisposition [5].

Bladder endometriosis is a rare site of the occurrence of endometriosis, in which the endometrial glands are present in the bladder wall. Within the bladder wall, the endometrial tissue infiltrates, typically at its base and dome. It often coexists with pelvic endometriosis, which leads to a variety of urinary and abdominal symptoms, including cyclical haematuria, dysuria, lower abdomen pain, and increased frequency [6]. The overall prevalence of endometriosis in the urinary tract is 1-2%, with the bladder as the most common site (84%) and urethral involvement as the second most common (15-20%).

Bladder endometriosis was first documented by Judd in 1921. It can be classified broadly into two types: primary bladder endometriosis occurring in women with no history of previous uterine surgery. Secondary bladder endometriosis develops following a prior history of gynaecological procedures such as caesarean sections.

Clinical features are cyclical haematuria, lower abdominal pain, and dysuria and can present with acute urinary retention. Haematuria is typical during menstruation, but cyclical haematuria occurs in only about 20-35% of women.

The gold standard for diagnosing bladder endometriosis is cystoscopy and biopsy, which allows direct visualisation of the bladder wall and biopsy of suspicious lesions. During cystoscopy, dark blue or reddish solid lesions are usually seen, surrounded by an oedematous halo or cystic formations. The appearance of lesions can vary in relation to the menstrual cycle in terms of size and pigmentation.

Treatment depends on several factors, including the size, location, and severity of the lesions. There is no definitive protocol currently for the management of bladder endometriosis. Initial treatment with hormonal therapy with progestogens, oral contraceptives or danazol is often the first step in managing symptoms. However, if medical treatment fails or symptoms do not resolve, surgical management may be necessary. Endoscopic resection is currently the preferred option because it is helpful as both a diagnostic and a therapeutic modality. In refractory cases with severe haematuria, partial cystectomy may be required [7].

Umbilical endometriosis, also called Villar's nodule, is a sporadic form of endometriosis. The incidence of umbilical endometriosis is 3.2-4.0% of patients presenting with endometriosis. It usually occurs due to iatrogenic seeding, where endometrial cells are deposited in the umbilicus during uterine surgery, commonly a caesarean section. However, umbilical endometriosis can rarely develop in women with no previous history of abdominal surgery (primary umbilical endometriosis). The exact pathogenesis of endometriosis is not fully understood. This is hypothesised to occur through blood or lymphatic spread of endometrial cells or through metaplasia of remnant urachal tissue in the umbilical tissue due to chronic inflammation [8].

Our patient had a previous caesarean section, making secondary umbilical endometriosis the most likely diagnosis. The incidence of secondary umbilical endometriosis is 1% among women who undergo a caesarean section, as the umbilicus serves as a physiological seeding site, leading to a higher incidence of umbilical endometriosis. Clinically, umbilical endometriosis often presents as a firm swelling at the umbilicus, which can be painful and, in some cases, exhibits cyclical bleeding during menstruation. Common symptoms include cyclical umbilical bleeding (49.2%), cyclical pain around the umbilicus (81.5%), and umbilical swelling (90.9%) [9].

Symptoms often persist for an average duration of one year. The condition is most commonly observed at the mean age of presentation of 37-45 years, particularly in the premenopausal age group.

Treatment is not standardised, and management usually depends on symptom severity and lesion extent. Endometriosis may generally respond to hormonal therapy but will require prolonged therapy for symptomatic control. The definitive treatment is usually surgical excision, which can be performed by total umbilical excision, involving the removal of the entire umbilicus, followed by reconstruction of the abdominal wall. Second is local resection, in which excision of only the endometriotic tissue is performed while preserving the umbilicus. In both approaches, removing the margin of normal tissue around the affected area is recommended to reduce the risk of recurrence [10].

This manuscript presents the first documented case in the literature of simultaneous bladder and umbilical endometriosis in the same patient, offering a unique and rare clinical insight into two individually uncommon manifestations of endometriosis. It highlights a multidisciplinary diagnostic approach involving imaging, cystoscopy, and histopathology, providing a structured evaluation pathway for atypical presentations.

The case holds strong clinical relevance across gynaecology, urology, radiology, and general surgery, expanding its interdisciplinary appeal. Notably, the patient was successfully managed with hormonal therapy, underscoring the value of conservative treatment in selected cases. By reinforcing the need to consider endometriosis in differential diagnoses of cyclical haematuria and umbilical bleeding, particularly in women with prior uterine surgery, this report adds both educational value and diagnostic awareness to existing literature. Bladder endometriosis should always be one of the differential diagnoses of haematuria with a previous history of gynaecological surgery. Not all endometriosis requires surgery; endometriosis can be managed initially with hormonal treatment as the first line of management.

## Conclusions

Understanding bladder and umbilical endometriosis is essential for the medical community due to their rarity, diagnostic challenges, and potential for misdiagnosis. These conditions may mimic more common urological conditions, leading to delayed or inaccurate treatment. Bladder endometriosis can present through cyclical haematuria and pelvic pain. Umbilical endometriosis, especially in the absence of a surgical history, may remain unrecognised. Recognising these clinical manifestations ensures timely intervention with appropriate hormonal or surgical management.

Furthermore, awareness promotes a multidisciplinary approach, bridging gynaecology, urology, radiology, and pathology for optimal care delivery. Early diagnosis and an individualised treatment approach can significantly improve patient outcomes and quality of life.
